# Supplementary material for: A Comprehensive Assessment of Ultraviolet-Radiation-Induced Mutations in Flammulina filiformis Using Whole-Genome Resequencing
Source: J Fungi (Basel). 2024 Mar 20;10(3):228. doi: 10.3390/jof10030228 (PMC10971301; doi:10.3390/jof10030228)
Supplement: Supplementary file 1 [file jof-10-00228-s001.zip › Supplementary Material S8/KEGG annotation/out/64381550635650.os/KO/out_map/map05152.html]

KEGG PATHWAY: Tuberculosis - Reference pathway


|  |  |
| --- | --- |
| **Tuberculosis - Reference pathway** |  |

[
Pathway menu
| Organism menu
| Pathway entry
| Show description
| User data mapping
]

Tuberculosis, or TB, is an infectious disease caused by Mycobacterium tuberculosis. One third of the world's population is thought to be infected with TB. About 90% of those infected result in latent infections, and about 10% of latent infections develop active diseases when their immune system is impaired due to the age, other diseases such as AIDS or exposure to immunosuppressive drugs. TB is transmitted through the air and primarily attacks the lungs, then it can spread by the circulatory system to other parts of body. Once TB bacilli have entered the host by the respiratory route and infected macrophages in the lungs, they interfere with phagosomal maturation, antigen presentation, apoptosis and host immune system to establish persistent or latent infection.

|  |  |
| --- | --- |
| Reference pathway | 100% |
